# Additively manufactured customized microhelix motors' bursting motion in mesoscopic tubes for vessel declogging†

**DOI:** 10.1039/d3ra07704g

**Published:** 2024-01-16

**Authors:** Yang Cao, Hongyu Yi, Kongyu Ge, Yifan Gao, Zhenchao Zhang, Huanhuan Feng

**Affiliations:** a Sauvage Laboratory for Smart Materials, Shenzhen Key Laboratory of Flexible Printed Electronics Technology, Harbin Institute of Technology (Shenzhen) China fenghuanhuan@hit.edu.cn +86755-86148426; b State Key Laboratory of Advanced Welding and Joining (Shenzhen), Harbin Institute of Technology (Shenzhen) China

## Abstract

Magnetic microhelix motors are widely employed in various applications such as cargo transportation, drug delivery, toxic substance declogging, and cell manipulation, due to their unique adaptive magnetic manipulation capabilities. In this work, high-precision stereoscopic additive manufacturing techniques were used to produce customized microhelices with varying structural parameters, including different pitch numbers (2–4 pitches), sizes (0.1–0.25 mm), and taper angles (172°–180°). Their motion performance in mesoscopic tubes was systematically investigated. The magnetic microhelix motors' speed increases when circle numbers and taper angles decrease, while circle diameters increase. The magnetic microhelix motors' speed could achieve a 1500% enhancement reaching 0.16 mm s^−1^ in a 0.3 mm tube, with a pitch number of 3, diameter of 0.2 mm, and taper angle of 172°. Furthermore, their vessel declogging capability is confirmed in *in vitro* experiments.

## Abstract 2

Magnetic microhelix motors are widely employed in various applications such as cargo transportation, drug delivery, toxic substance declogging, and cell manipulation, due to their unique adaptive magnetic manipulation capabilities.

## Graphical abstract

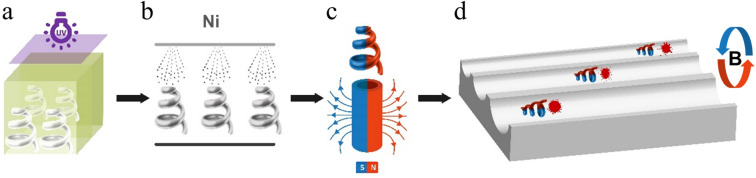

## Introduction

Microhelix motors are fascinating artificial tinny machines.^1–11^ They are capable of autonomous motion-powered tasks,^12–15^ such as target drug delivery,^16,17^ microsurgery,^18,19^ biosensing,^20–22^ and contaminant degradation.^23,24^ In general, motors based on varying materials and structures gain thrust from either chemical reactions or external fields. Chemical-driven motors transfer the chemical energy stored in fuels to driven power, but chemical reactions are easily affected by complex chemical environments in the human body, thereby reducing stability.^25^ Magnetic propulsion is another common way for micromotor movement. By featuring micromotor magnetism, they can be easily manipulated through different applied magnetic fields, without interacting with surroundings. This guarantees their great stability and accuracy. Therefore, magnetic micromotors have been widely studied in recent years.^26,27^

The fabrication of magnetic microhelix motors is based on the most advanced materials science and engineering technology and combined with multidisciplinary knowledge (bioengineering, computer science, mechanical engineering).^27–33^ Four fabrication methods are mainly employed to make micro and nano helical structures, namely rolled-up, GLAD (Glancing Angle Deposition), DLW (Direct Laser Writing), and TA (Template-Assisted).^34–40^ Huang's team used the Rolled-up method to fabricate reconfigurable micro-origami motors.^41^ This method is the most convenient way for the mass production of micromotors and is capable of fabricating motors with any inorganic materials.^42^ However, it can merely fabricate tubular motors, which limits the motor geometry. DLW is a powerful method allowing the fabrication of designable and complex nano-scale structures with high resolution.^43^ This technique is quite flexible, but the complex procedures result in high costs and hinder the mass-production. GLAD is a useful tool for nanofabrication, which can deposit any type of structure with almost no material limit.^44,45^ Fischer's team fabricated one of the smallest magnetically driven nano screws with a diameter of 70 nm using the GLAD method.^46^ While GLAD has proved to be a very promising technique for nanostructure fabrication, the exclusive machines and complex procedures are still crucial challenges to be solved. Template-based methods are low-cost, convenient ways to fabricate versatile micromotors, but they are not sophisticated enough to control the growth orientations.^47^

Additive manufacturing (or 3D printing) has been proven to be a promising nanofabrication method in recent years.^48^ The designed 3D objects are realized by adding materials layer by layer, which minimizes waste. Projection micro stereolitho-graphy is a new additive manufacturing method based on ultraviolet light polymerization. The 3D model will be sliced into several 2D patterns in advance, and the shaped ultraviolet light with the corresponding patterns will be focused on the prepolymer. Each exposure will be able to fabricate one layer of the object, which enables high-precision, low-cost, and convenient nanofabrication.^49^

**Fig. 1:**
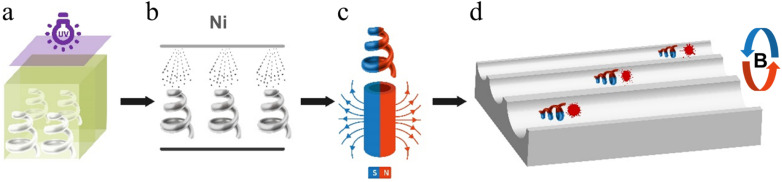
Schematic diagram of magnetic microhelix motor fabrication and declogging experiment. (a) Additive manufacture. (b) Ni sputtering. (c) Polarity distribution. (d) Moving performance tests.

This work successfully fabricates microhelix motors with different structures using a high-precision additive manufacturing device, BMF S130. It can create complex 3D objects with exceptional precision, down to 2 μm, by layering materials to construct designed structures. Its capability provides a powerful toolset for systematic additive manufacturing of microhelices. The fabrication process is shown in Fig. 1a and b. The magnetic response can be featured *via* sputtering Ni, and the magnetic pole distribution is oriented longitudinally along the rotational axis of the microhelices. Microhelix can be axially rotated under the magnetic field and the microhelix motors can be easily manipulated for different functions, rendering it an ideal candidate for simulating *in vitro* embolus declogging.

## Materials and methods

### Materials

Demi-water, anhydrous ethanol, sodium dodecyl sulfate (Macklin's), HTL-type light-curing resin (Shenzhen BMF Co., Ltd), and other chemicals are primarily used during the experimental operation. A 0.9% concentration saline solution is used in the investigation of the motion law of the magnetic microhelix motor.

### Microhelix motor fabrication

High-precision stereoscopic additive manufacturing is achieved using a BMF S130 printer, which has an additive manufacturing accuracy of 2 microns, a maximum format of 50 mm (*L*) * 50 mm (*W*), and a print layer thickness of 5 μm. The printing solution is HTL-type light-curing resin. Motors with different parameters are designed using SolidWorks 3D modeling software. These models are sliced into layers with a thickness of 5 μm each. The sliced data is then imported into the 3D printer. The UV light intensity is set at 70 mW cm^−2^, and each layer's leveling time ranges from 90 seconds to 150 seconds. Additionally, a scraper moves from left to right to remove any bubbles every 6 layers, preventing possible negative effects.

### Magnetism featuring

Magnetic response is featured *via* sputtering Ni. A vacuum nickel-plating machine is the primary instrument used to confer magnetism to the microhelix. The sputtering parameters are as follows: the atmospheric pressure is 4 × 10^−4^ Pa, the sputtering rate is around 0.3 Å s^−1^, and the thickness is around 600 Å when the sputtering time is 30 minutes.

### Magnetic field preparation and driven

The magnetic field is created by a three-dimensional magnetic control device consisting of Helmholtz coils and Maxwell coils. Maxwell coils generate a gradient field, while Helmholtz coils generate a uniform magnetic field, thereby allowing for the translational motion in all directions of microhelix motors.

## Results

**Fig. 2:**
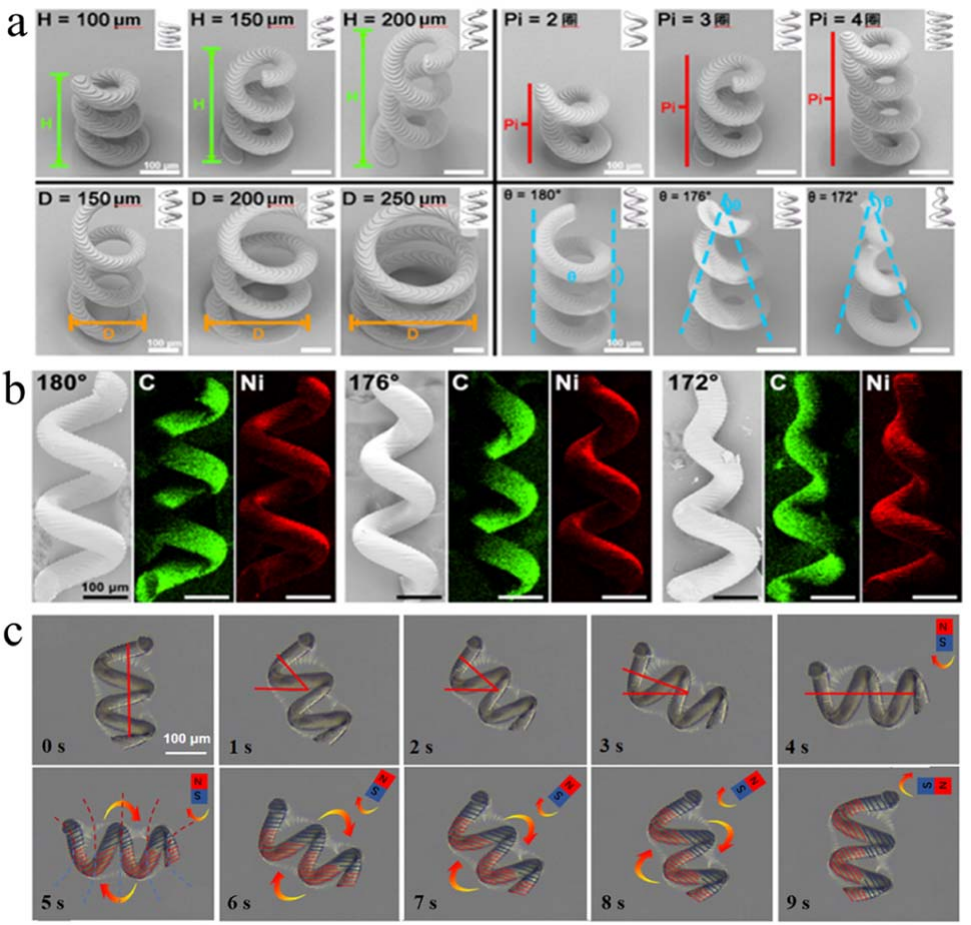
Magnetic microhelix characterization and magnetic field distribution. (a) Photomicrograph of magnetic microhelix. (b) EDS elemental analysis. (c) Verification of microhelix magnetic field distribution.

Scanning electron microscopy (SEM) is employed to validate the morphological and structural precision of the printed microhelices. The surface projection micro-stereolithography S130 system permits full customization of microhelix parameters at the micron scale, achieving an exceptional processing precision of up to 10 μm, as shown in Fig. 2a. This enables meticulous adjustment of distinct structural parameters and the control of dimensional error within a 10 μm margin.

Parameters such as microhelix height (*H*), pitch number (Pi), bottom circle diameter (*d*), and taper angle (*θ*) are investigated to optimize its moving and declogging capabilities. Microhelices with diverse parameters are manufactured, including height from 0.1 mm to 0.2 mm, pitch numbers from 2 to 4, bottom circle diameters from 0.1 mm to 0.2 mm, and taper angles varying between 180° and 172°. These microhelices are characterized in Fig. 2a.

To confer magnetic properties on those microhelices, a surface-sputtering process with nickel is employed. Microhelices characterized by taper angles of 180°, 176°, and 172° are chosen for Energy Dispersive Spectroscopy (EDS) elemental analysis after the nickel sputtering, as shown in Fig. 2b. In the EDS analysis, carbon is depicted in green, while nickel is depicted in red, indicating the successful deposition of metallic nickel on the microhelix surface.

Subsequently, the polarity distribution of the nickel-coated microhelix motor is investigated using a permanent magnet with a known polarity distribution. The magnet is initially positioned perpendicular to the motor and moves toward it. The motor responds with a 90° clockwise rotation, aligning itself in parallel with the magnet. Furthermore, the motor exhibits synchronized rotation with the permanent magnet when the latter is rotated clockwise. Upon the cessation of the magnet's rotation, the motor also comes to a stop, maintaining its parallel alignment with the magnet, as shown in Fig. 2c. These observations suggest that the microhelix motor possesses a transverse polarity distribution, consistent with the polarity distribution of the permanent magnet employed in this investigation.

The optimization of microhelix motors is realized through the manipulation of key parameters, including pitch number (Pi), motor diameter (*d*), tube diameter (*D*), and taper angle (*θ*). Comparative speed experiments are conducted for magnetic microhelix motors in both constrained and non-constrained environments. These motors are principally propelled by a three-dimensional magnetic field, which is generated by Maxwell and Helmholtz coils. Therefore, they can attain planar rotational forward motion under controlled conditions.

**Fig. 3:**
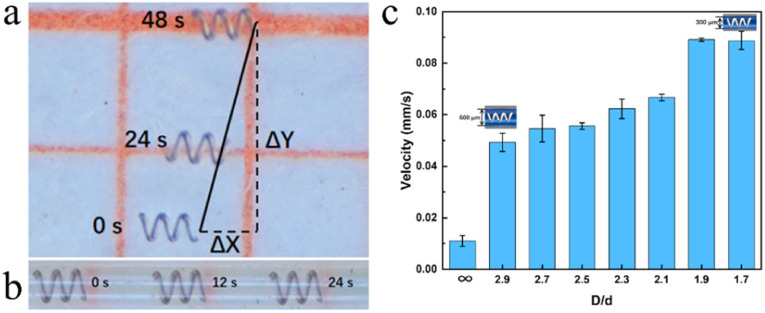
The swimming performance in different constrained areas. (a) Motor motion in the non-constrained area. (b) Motor motion in the constrained area. (c) Swimming velocity in various constrained areas.

Microhelix motors with specific parameters – a pitch number (Pi) of 3, a taper angle (*θ*) of 180°, and a bottom circle diameter (*d*) of 0.2 mm – are chosen for the investigation of their performance under a controlled Helmholtz magnetic field frequency of 10 Hz. The motion speeds are recorded in both non-constrained and constrained areas, as detailed in Fig. 3 and Table 1. The speed of the microhelix motor in the constrained area exceeds that in the non-constrained area by a factor of 4. The details are shown in videos S1 and S2.†

**Table 1:** Comparison of micro-helix motion in constrained (C) and non-constrained (NC) environments

Direction of motion	Displacement (mm)		Average speed (mm s^−1^)	
	NC	C	Non-constrained environment	Constrained environment
*Y*-direction	1.9	0	0.079	0
*X*-direction	0.5	2	0.021	0.083

To investigate the influence of the diameter of tubes, mesoscopic tubes ranging from 0.3 mm to 0.6 mm in diameter (*D*) are fabricated for subsequent experiments. The tubes are filled with a 0.9% saline solution to offset the gravitational effects on the microhelix motor. Microhelix motors are placed inside the mesoscopic tubes with varying diameters under a controlled magnetic field of 10 Hz. Motion analysis is conducted using high-speed, high-definition digital cameras, and forward speed is measured using the picture interception method. The results are shown in Fig. 3c. The microhelix motor's speed displays a positive correlation with the *D*/*d* ratio, suggesting that increased confinement within the mesoscopic tube leads to enhanced forward speed by minimizing lateral drift.

**Fig. 4:**
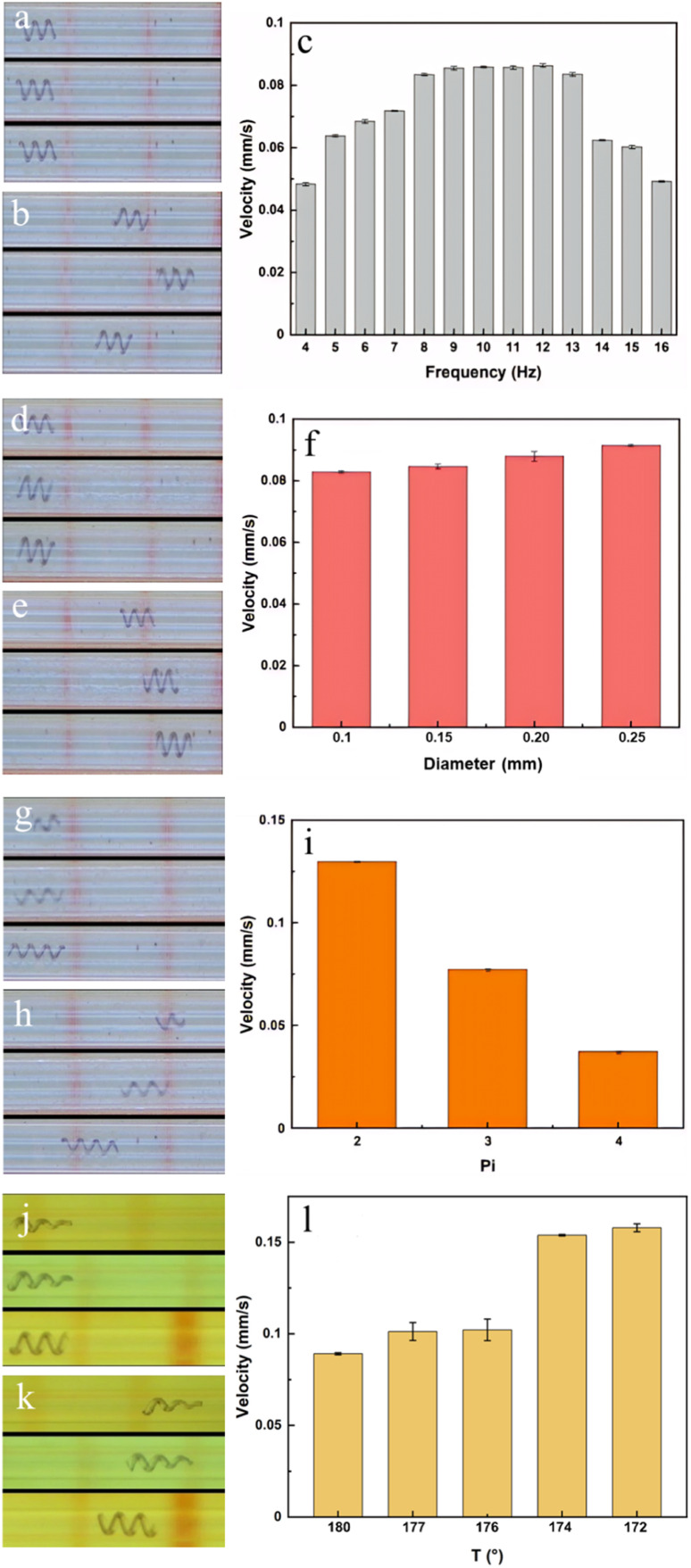
Swimming velocity tests under different conditions. (a) and (b) Moving performance under a 6, 10, and 15 Hz magnetic field. (c) Swimming velocity under varying magnetic fields. (d) and (e) Moving performance of motors with 0.15, 0.2, and 0.25 mm bottom diameters. (f) Swimming velocity of motors with varying bottom diameters. (g) and (h) Moving performance of motors with 2, 3, and 4 pitch numbers. (i) Swimming velocity of motors with varying pitch numbers. (j) and (k) Moving performance of motors with 172°, 176°, and 180° taper angles. (l) Swimming velocity of motors with varying taper angles.

Subsequently, the correlation between the forward motion speed of the microhelix motor and the magnetic field is examined. The frequency of magnetic field alterations plays a crucial role in determining the motor's performance. Thereby, a motor with a 0.2 mm bottom diameter, 3 pitches, and a 180° taper angle is selected and placed inside a rigid glass tube with a 0.3 mm inner diameter for frequency experiments. The motor is subjected to varying magnetic field frequencies, and the swimming velocity is recorded, as shown in Fig. 4a–c and video S3.† When the magnetic field frequency is set to 9, 10, 11, or 12 Hz, the velocity of the motor reaches its maximum. This is because a low-frequency magnetic field provides insufficient energy for motor movement, while motors cannot keep up with a magnetic field of high frequency. Therefore, a frequency of 10 Hz is selected for the following tests.

Motors with different parameters are fabricated to identify the highest velocity. The details are shown in Table 2. Motors in groups 1, 2, 3, and 4 are chosen to investigate the influence of varying bottom diameters. The results are shown in Fig. 4d–f and video S4.† The results show that as the bottom diameter is increased from 0.1 mm to 0.25 mm, the speed is slightly raised from 0.08 mm s^−1^ to 0.09 mm s^−1^, representing a 12% enhancement. However, a large bottom diameter may result in motors becoming lodged in veins, impeding their forward movement; and the clogs also may pass through the motor without being effectively pushed. Therefore, motors with diameters of 0.2 mm are selected for the following tests.

**Table 2:** Parameters of different motors

Group	Bottom diameters (mm)	Pitch numbers	Tapers (°)
1	0.1	3	180
2	0.15	3	180
3	0.2	3	180
4	0.25	3	180
5	0.2	2	180
6	0.2	4	180
7	0.2	3	177
8	0.2	3	176
9	0.2	3	174
10	0.2	3	172

Subsequently, motors in groups 3, 5, and 6 are chosen to investigate the influence of different pitch numbers. The results are shown in Fig. 4g–i and video S5.† As the pitch number increases, the motor speed experiences a significant reduction. When the number of motor pitches is reduced from 4 to 2, the motor speed increases from 0.04 mm s^−1^ to 0.13 mm s^−1^, representing a 225% enhancement. However, motors with pitch numbers below 3 posed challenges in controlling their direction of movement. Thereby, motors with 3 pitches are chosen for the following tests.

Finally, motors in groups 3, 7, 8, 9, and 10 are chosen to explore the impact of different taper angles. The results are shown in Fig. 4j–l and video S6.† Altering the taper angle from 180° to 172° leads to an increase in motor speed, rising from 0.08 mm s^−1^ to 0.16 mm s^−1^, representing a 100% enhancement. Meanwhile, motors with a 172° taper angle demonstrate the ability to prevent clogs from passing through.

**Fig. 5:**
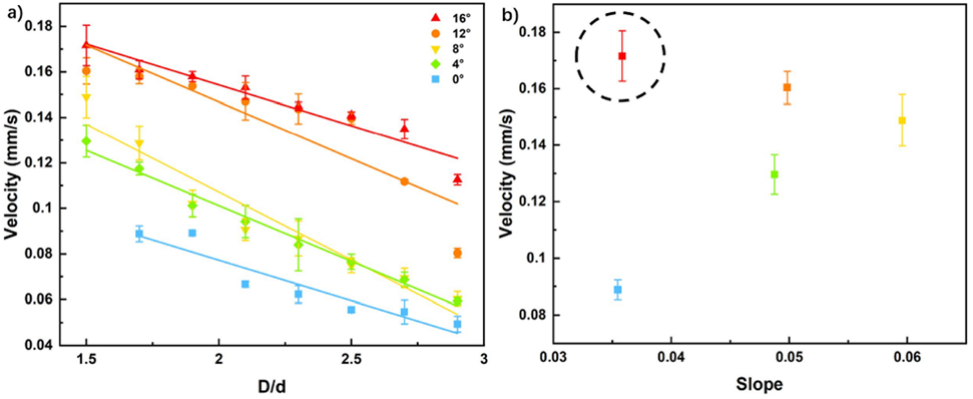
Comparison of tapered magnetic micromotors under constrained areas. (a) Relationship between *D*/*d* and the forward speed of the motor with different tapers; (b) optimal values of motor speed.

Now, we've demonstrated the motor speed could be lifted with a constrained area, moderate magnetic frequency, smaller bottom diameter, fewer pitch numbers, and reduced taper angles. Constrained area limits the motor's lateral moving, fewer pitch numbers reduce the rotary energy the motor needs, and reduced taper angles give the motor smaller viscous force. Fig. 5 summarizes the constrained area and taper angle factors. As the taper angle decreases, the motor speed increases, and the relative increase in speed reaches the greatest at 12°. However, the highest absolute speed of the motor is at 16° with the smallest *D*/*d*. Therefore, through the experiments described above, optimal parameters are gained: a bottom diameter of 0.2 mm, a pitch number of 3, and a taper angle of 172°, corresponding to motors from group 10.

**Fig. 6:**
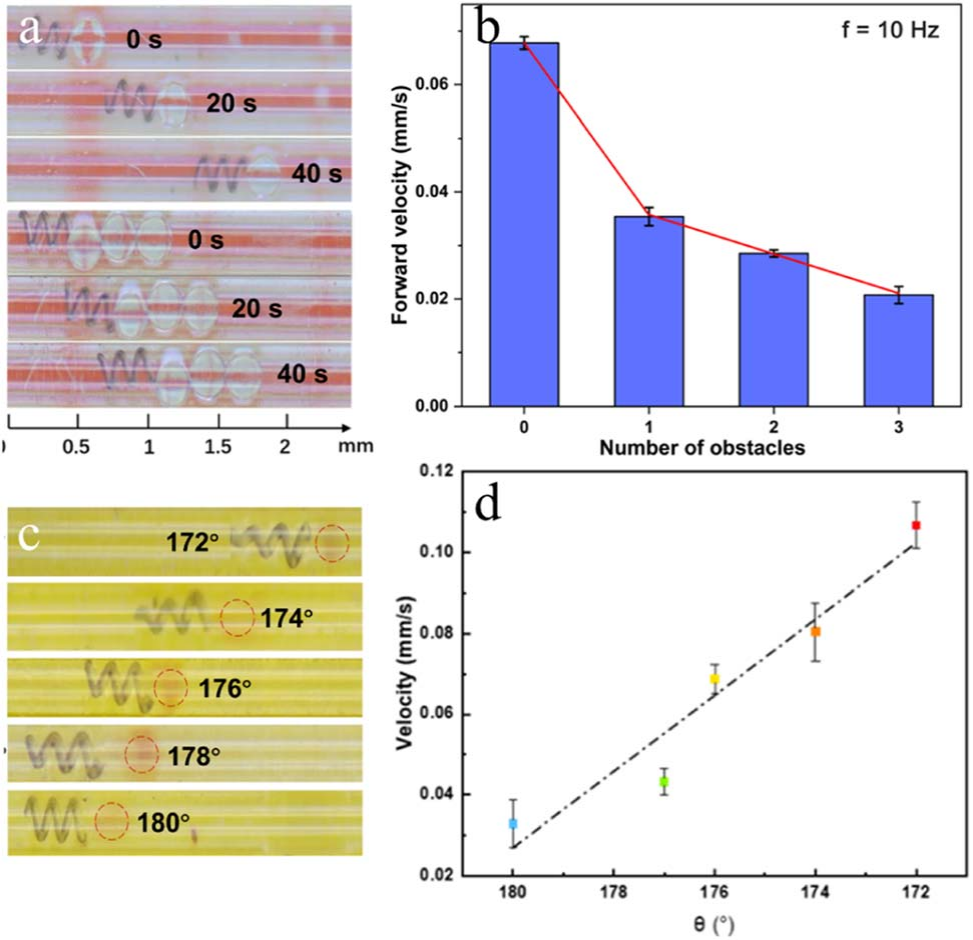
Arterial vascular embolism push experiment. (a) The moving performance of motors pushes 1 and 3 rigid balls. (b) Swimming velocity of motors pushing different numbers of balls. (c) Motors push clogs with different taper angles. (d) Swimming velocity of motors with varying taper angles during push experiments.

The optimized microhelix motor is subsequently subjected to obstacle-pushing experiments, during which it encounters and pushes varying numbers of polystyrene (PS) balls (0, 1, 2, and 3), each with an approximate diameter of 0.3 mm and a mass of 0.06 g. The experiments are conducted in tubes with a diameter of 0.4 mm, filled with a 0.9% saline solution to offset the gravitational effects on the microhelix motor, as shown in Fig. 6a and video S7.† Motion analysis is conducted using high-speed, high-definition digital cameras, and forward speed is measured using the picture interception method on multiple occasions. The results are illustrated in Fig. 6b. Maintaining a constant mesoscopic tube diameter, we observe a decline in motor speed as the number of PS balls increases. When the number of PS balls reaches three, the speed decreases to approximately 0.02 mm s^−1^, effectively reaching the magnetic microhelix motor's maximum carrying capacity. These experiments demonstrate the motor's ability to transport rigid obstacles six times its mass at a speed of 0.02 mm s^−1^.

Subsequently, an arterial vascular embolism push experiment is conducted. In previous experiments, an intriguing phenomenon was observed: motors with taper angles exhibited different speeds during forward and backward motion. The motors exhibit higher speed when the larger end led, achieving an approximately 50% improvement compared to when the smaller end led. Furthermore, when these motors are pushing soft obstructions such as coagulated pig blood, the smaller-end-lead method may make the end lodged in the clog, while the larger-end-lead method allows for a smooth push, as shown in video S8.† Therefore, the larger end is chosen to take the lead during the experiments.

Microhelix motors, featuring optimal performance parameters as determined from prior experimental studies, are chosen and positioned within a simulated blood vessel to facilitate the removal of embolic materials. The mesoscopic tube has a diameter of 0.3 mm. To simulate arterial emboli, semi-solids created from coagulated pig blood are employed, while a 0.9% saline solution is used to replicate the internal environment of blood vessels. The influence of varying taper angles on the declogging process is investigated. Microhelix motors with a bottom diameter of 0.2 mm, featuring 3 pitches and varying tapers are chosen for the experiments. Blood clots are meticulously crafted into irregular shapes, each approximately 0.15 mm in diameter. A mesoscopic tube with a diameter of 0.3 mm is filled with a 0.9% saline solution to mimic the biological internal environment. The microhelix motors and blood clots are placed inside the tube, and the assembly is then positioned on the magnetic control platform. A consistent magnetic field of 10 Hz is applied to drive the magnetic microhelix motor, pushing the clot and simulating the thrombus declogging process. As shown in Fig. 6c and d, the microhelix motor's clot-pushing speed increases from 0.03 mm s^−1^ to 0.11 mm s^−1^, representing an approximate 200% increase, as the taper reduces from 180° to 172°. Consequently, the optimized magnetic microhelix motor demonstrates significantly greater efficiency in removing clot material.

## Conclusions

Additive manufacturing has been a versatile tool for the fabrication of complex customized microhelix motors. In this work, customized micromotors with adjustable structures are fabricated using an S130 3D printer, and the factors affecting the motors' moving performance are investigated. The motor speed can be lifted with a constrained area, moderate magnetic frequency, smaller bottom diameter, fewer pitch numbers, and reduced taper angles. The motor with a bottom diameter of 0.2 mm, a pitch number of 3, and a taper angle of 172° shows the greatest speed, reaching 0.16 mm s^−1^ under a constrained area with an applied 10 Hz magnetic field. Our micromotors outperform other motors of the same type and enhance the forward-moving speed 2–3 times (Table 3). The optimized motor is then applied in the *in vitro* vascular embolism push experiment. The customized motor exhibits great declogging performance for both rigid and soft obstacles.

**Table 3:** Performance comparison of related micromotors ^50–52^

References	Forward speed
50	64.75 μm s^−1^
51	86 μm s^−1^
52	Near 68 μm s^−1^
This work	160 μm s^−1^

## Author contributions

Yang Cao: formal analysis, investigation, writing – original draft. Hongyu Yi: investigation, writing – original draft, review & editing. Kongyu Ge: data curation, writing – review & editing. Zhenchao Zhang: investigation, data curation. Huanhuan Feng: conceptualization, methodology, writing – review & editing, supervision, project administration, funding acquisition.

## Conflicts of interest

The authors declare no conflict of interest.

## Supplementary Material

RA-014-D3RA07704G-s001

RA-014-D3RA07704G-s002

RA-014-D3RA07704G-s003

RA-014-D3RA07704G-s004

RA-014-D3RA07704G-s005

RA-014-D3RA07704G-s006

RA-014-D3RA07704G-s007

RA-014-D3RA07704G-s008
